# Supplementary material for: Slow conformational changes in the rigid and highly stable chymotrypsin inhibitor 2
Source: Protein Sci. 2023 Apr 1;32(4):e4604. doi: 10.1002/pro.4604 (PMC10031225; doi:10.1002/pro.4604)
Supplement: Supplementary file 1 — Data S1. Supporting information including nine figures with relaxation dispersion profiles for all residues at all conditions (Figure S1–S9), one plot of ΔG versus temperature for the N to N* transition (Figure S10) and a plot of the chemical shift differences from the relaxation dispersion measurements versus the chemical shift differences expected for complete unfolding (Figure S11). In addition, the supplementary material contains one table with a list of structures of CI2 (Table S1) and supplementary references. [file PRO-32-e4604-s001.pdf]

# SUPPLEMENTARY MATERIAL

## Slow conformational changes in the rigid and highly stable chymotrypsin inhibitor 2

Yulian Gavrilov[1], Andreas Prestel, Kresten Lindorff-Larsen and Kaare Teilum*

Structural Biology and NMR Laboratory, Linderstrøm-Lang Centre for Protein Science, Department of Biology, University of Copenhagen, Copenhagen N, Denmark

[1]Present address: Division of Biophysical Chemistry, Center for Molecular Protein Science, Department of Chemistry, Lund University, Lund, Sweden

*Correspondence: kaare.teilum@bio.ku.dk

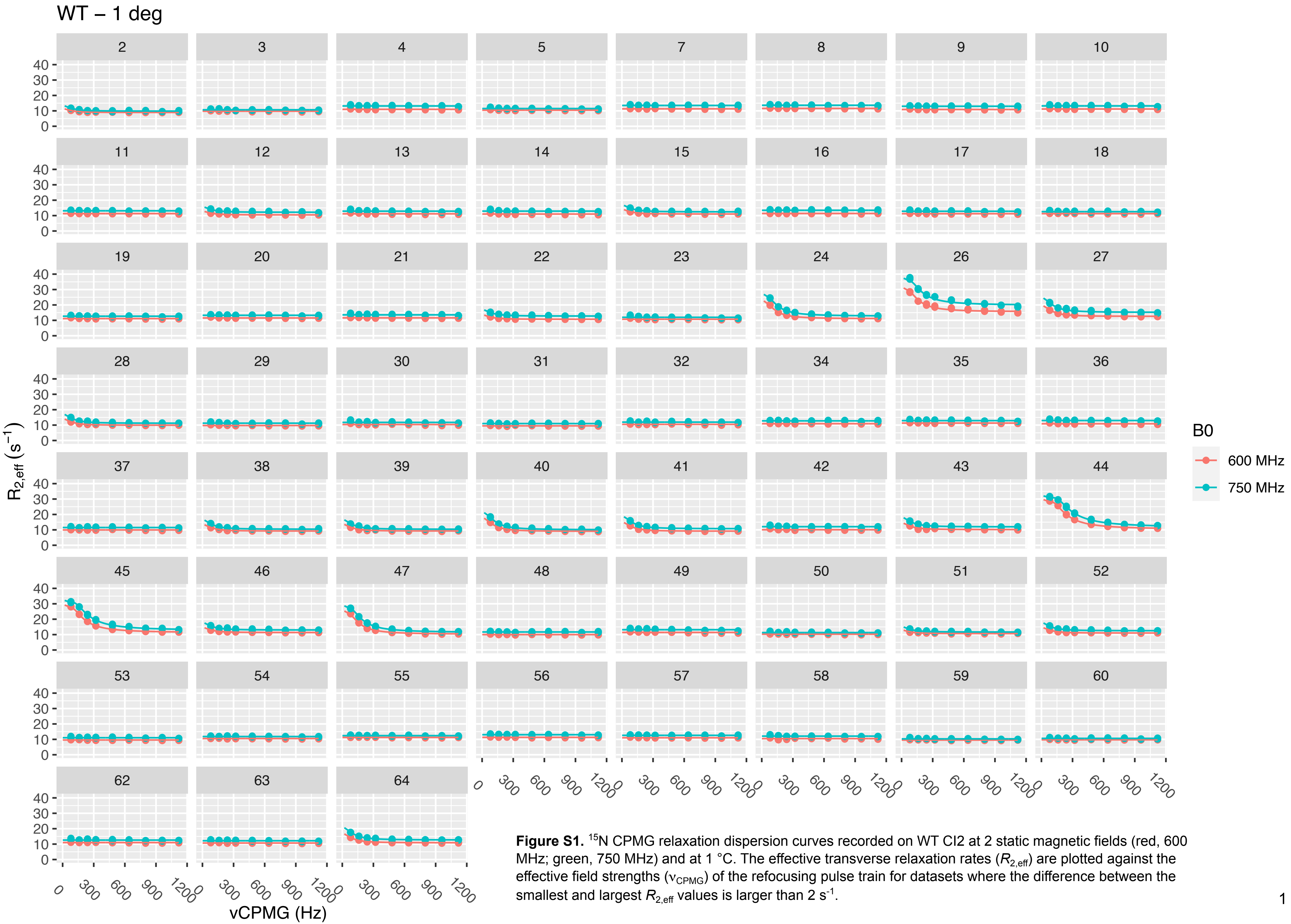


**Figure S1.** $^{15}$N CPMG relaxation dispersion curves recorded on WT CI2 at 2 static magnetic fields (red, 600 MHz; green, 750 MHz) and at 1 °C. The effective transverse relaxation rates ($R_{2,eff}$) are plotted against the effective field strengths ($\nu_{CPMG}$) of the refocusing pulse train for datasets where the difference between the smallest and largest $R_{2,eff}$ values is larger than 2 $s^{-1}$.

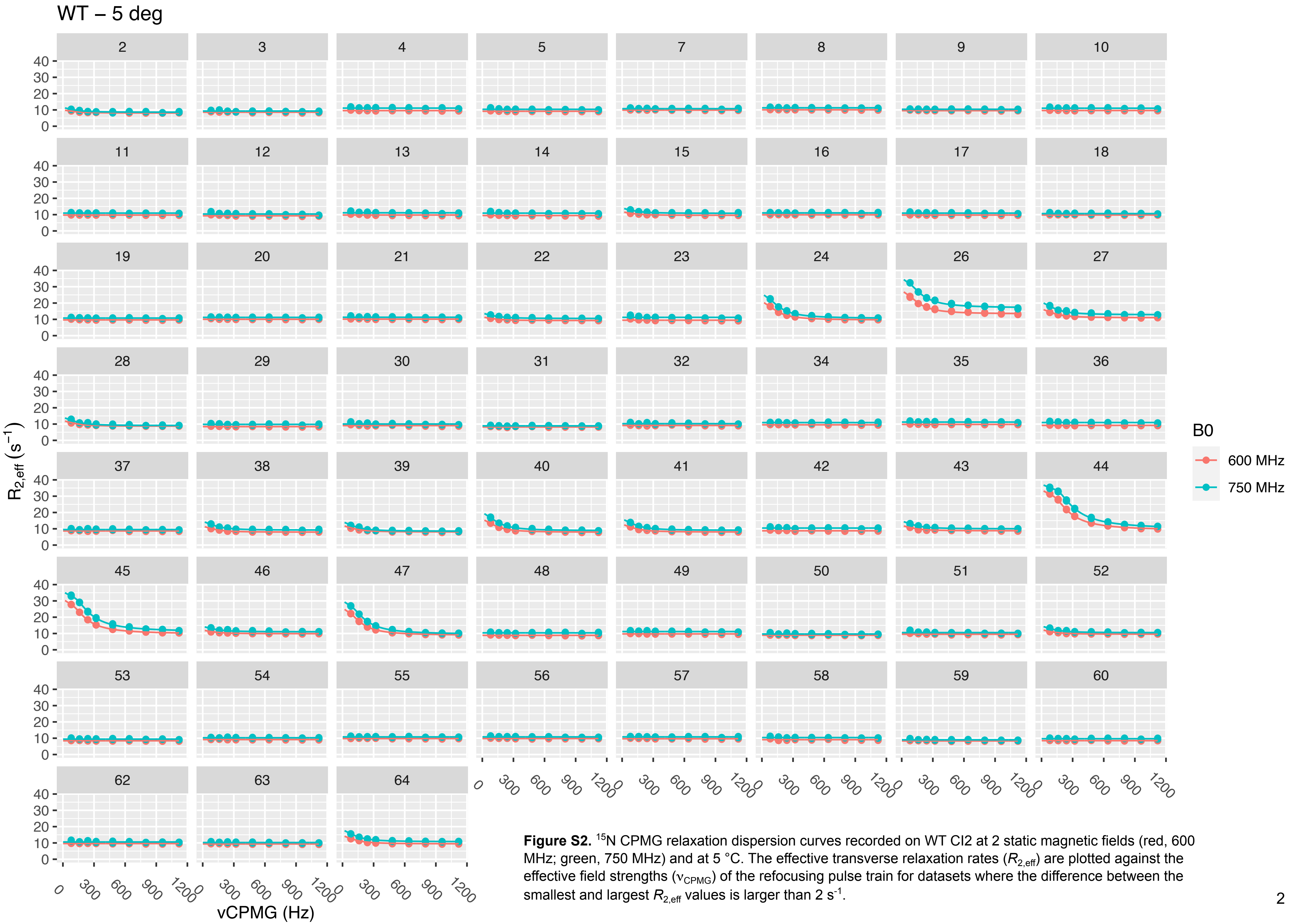


**Figure S2.** $^{15}$N CPMG relaxation dispersion curves recorded on WT CI2 at 2 static magnetic fields (red, 600 MHz; green, 750 MHz) and at 5 °C. The effective transverse relaxation rates ($R_{2,\mathrm{eff}}$) are plotted against the effective field strengths ($\nu_{\mathrm{CPMG}}$) of the refocusing pulse train for datasets where the difference between the smallest and largest $R_{2,\mathrm{eff}}$ values is larger than 2 $s^{-1}$.

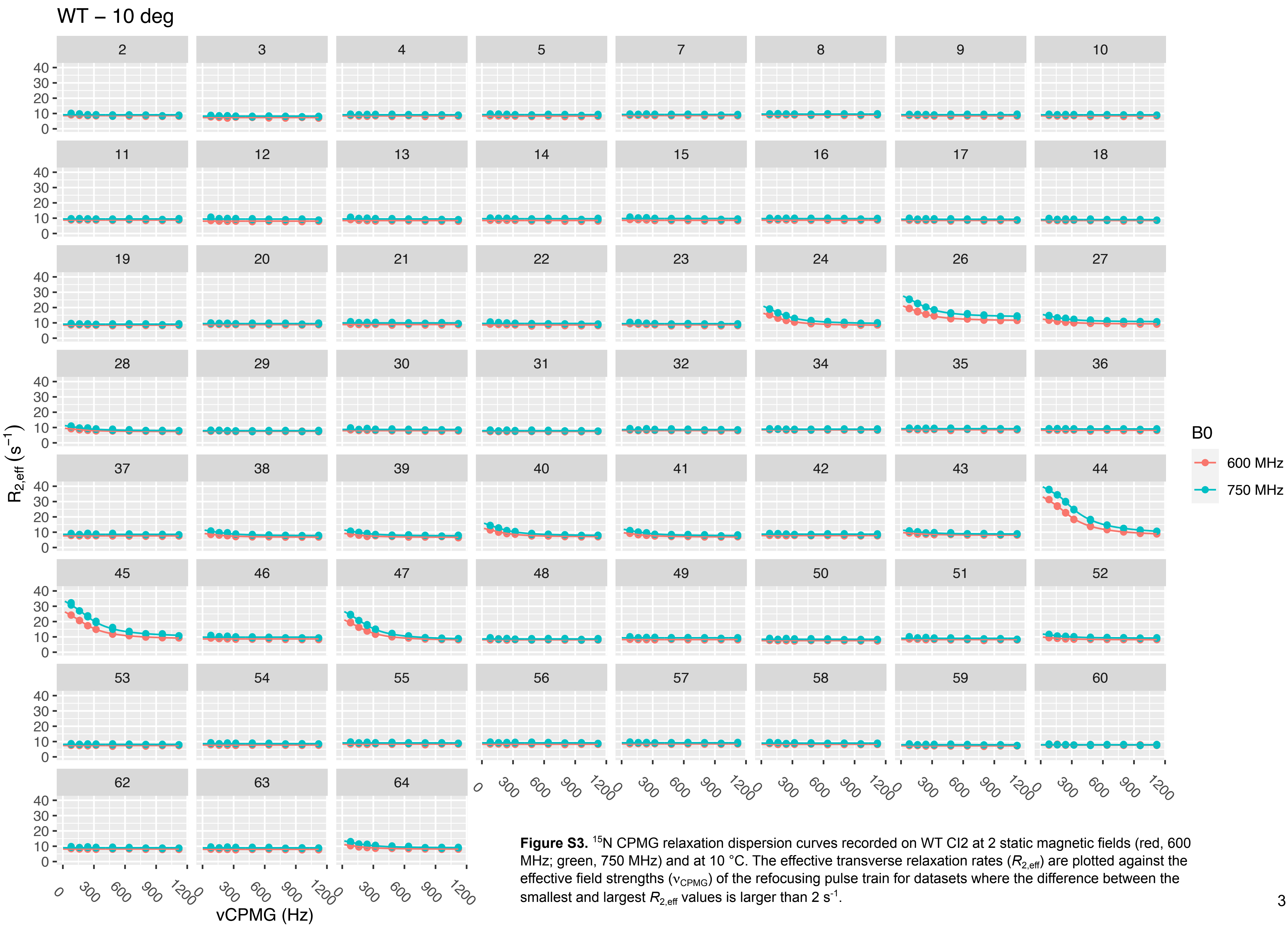


**Figure S3.** $^{15}N$ CPMG relaxation dispersion curves recorded on WT CI2 at 2 static magnetic fields (red, 600 MHz; green, 750 MHz) and at 10 °C. The effective transverse relaxation rates ($R_{2,eff}$) are plotted against the effective field strengths ($\nu_{CPMG}$) of the refocusing pulse train for datasets where the difference between the smallest and largest $R_{2,eff}$ values is larger than 2 $s^{-1}$.

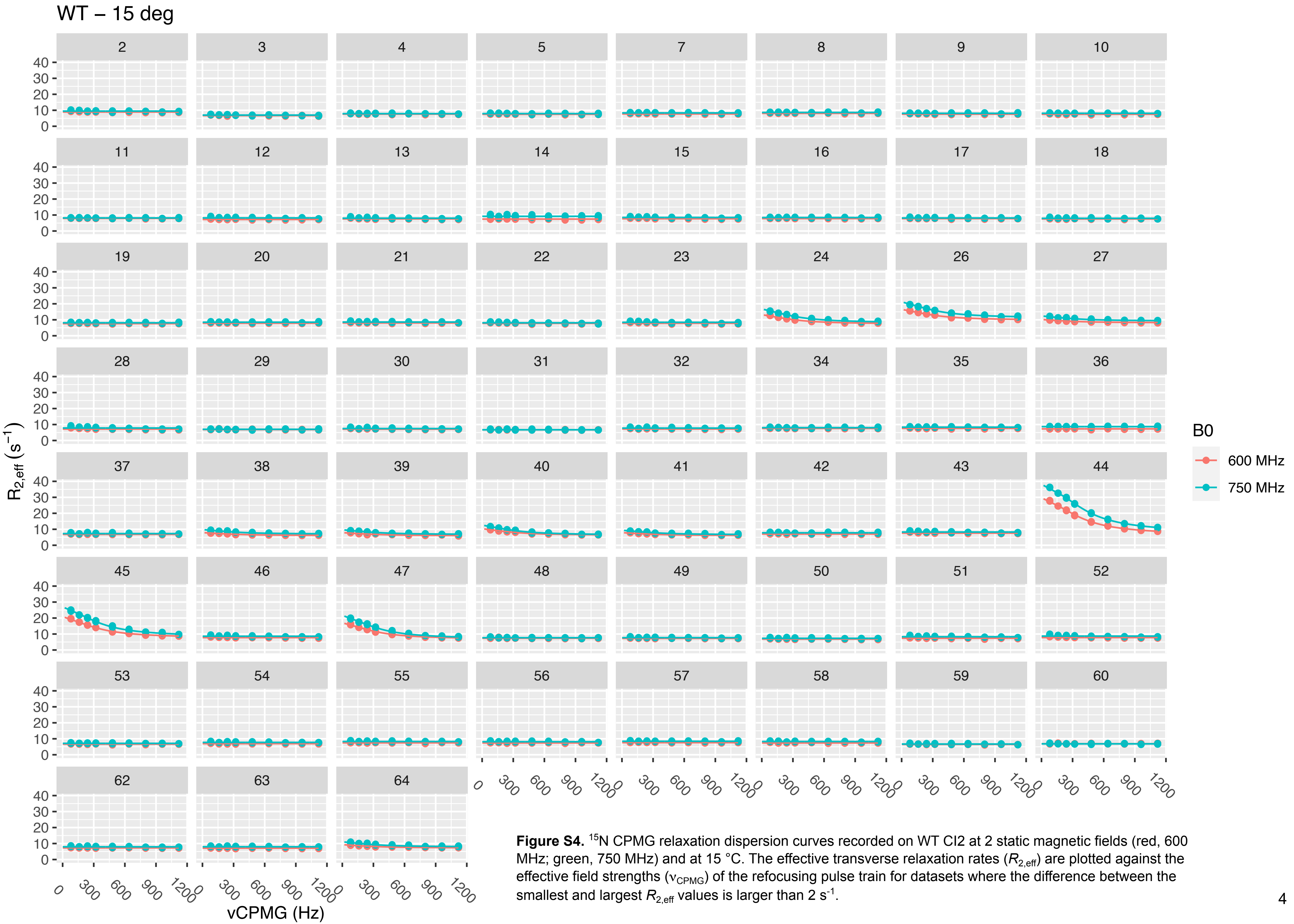


**Figure S4.** $^{15}$N CPMG relaxation dispersion curves recorded on WT CI2 at 2 static magnetic fields (red, 600 MHz; green, 750 MHz) and at 15 °C. The effective transverse relaxation rates ($R_{2,\mathrm{eff}}$) are plotted against the effective field strengths ($\nu_{\mathrm{CPMG}}$) of the refocusing pulse train for datasets where the difference between the smallest and largest $R_{2,\mathrm{eff}}$ values is larger than 2 $s^{-1}$.

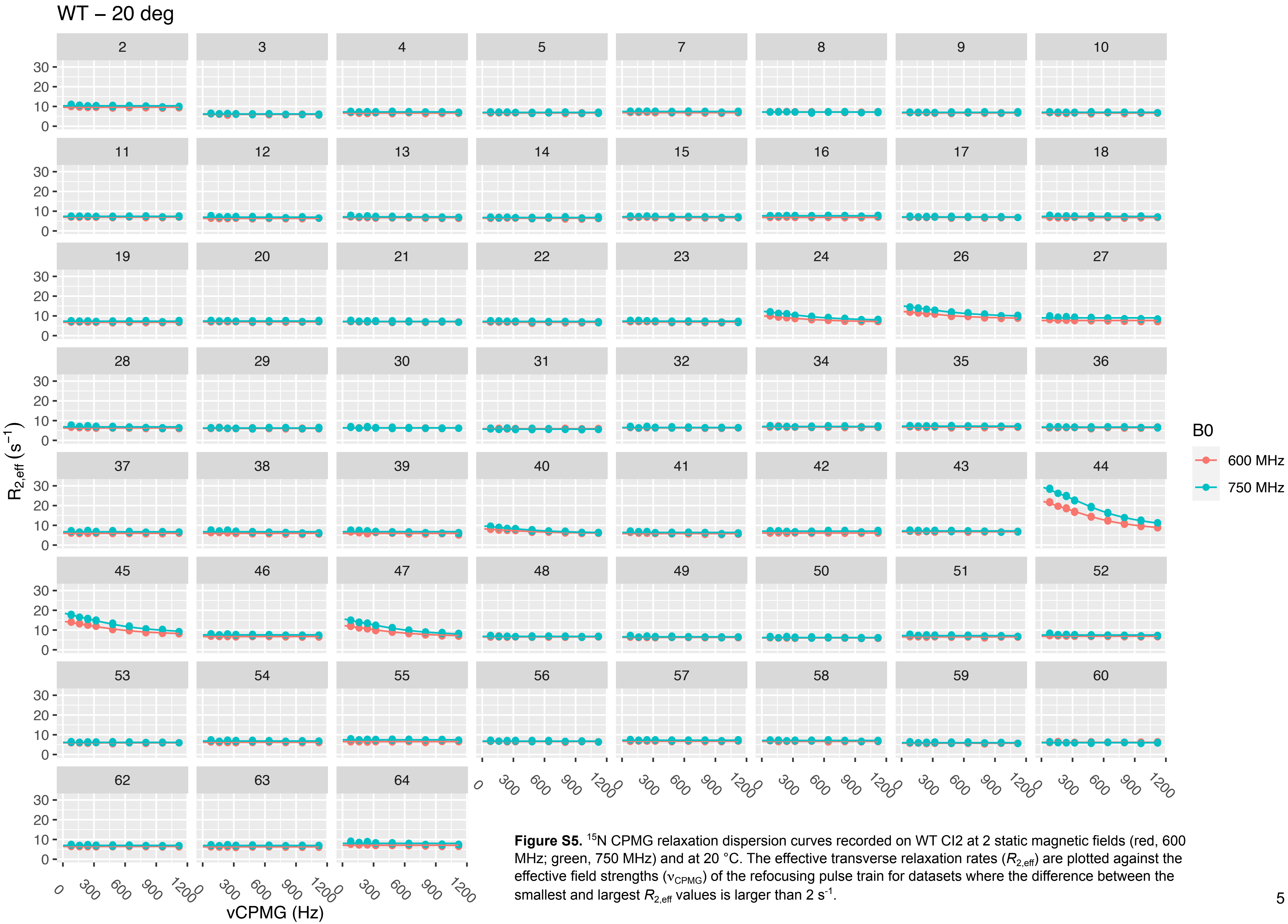


**Figure S5.** $^{15}$N CPMG relaxation dispersion curves recorded on WT CI2 at 2 static magnetic fields (red, 600 MHz; green, 750 MHz) and at 20 °C. The effective transverse relaxation rates ($R_{2,eff}$) are plotted against the effective field strengths ($\nu_{CPMG}$) of the refocusing pulse train for datasets where the difference between the smallest and largest $R_{2,eff}$ values is larger than 2 $s^{-1}$.

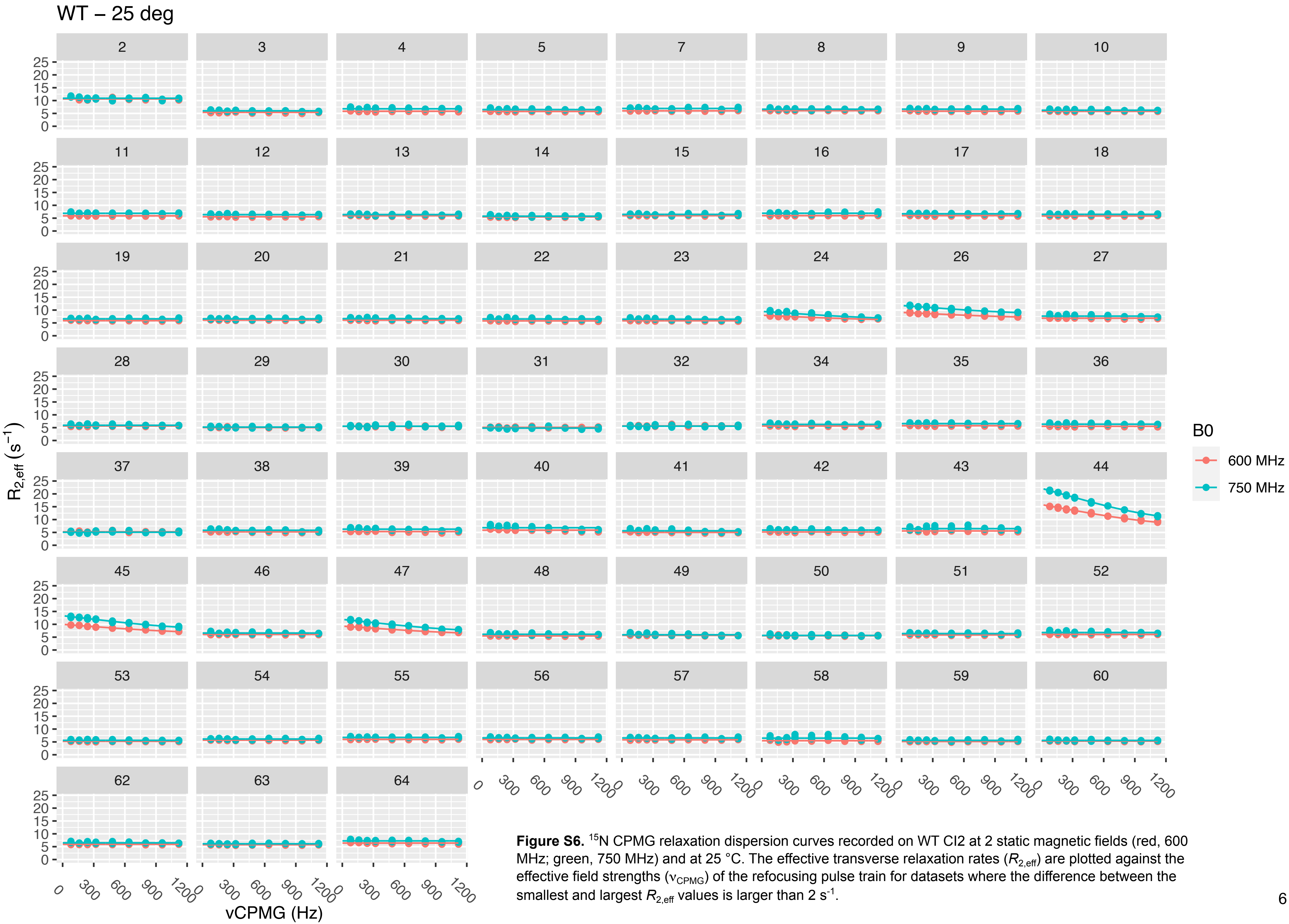


**Figure S6.** $^{15}$N CPMG relaxation dispersion curves recorded on WT CI2 at 2 static magnetic fields (red, 600 MHz; green, 750 MHz) and at 25 °C. The effective transverse relaxation rates ($R_{2,eff}$) are plotted against the effective field strengths ($\nu_{CPMG}$) of the refocusing pulse train for datasets where the difference between the smallest and largest $R_{2,eff}$ values is larger than 2 $s^{-1}$.

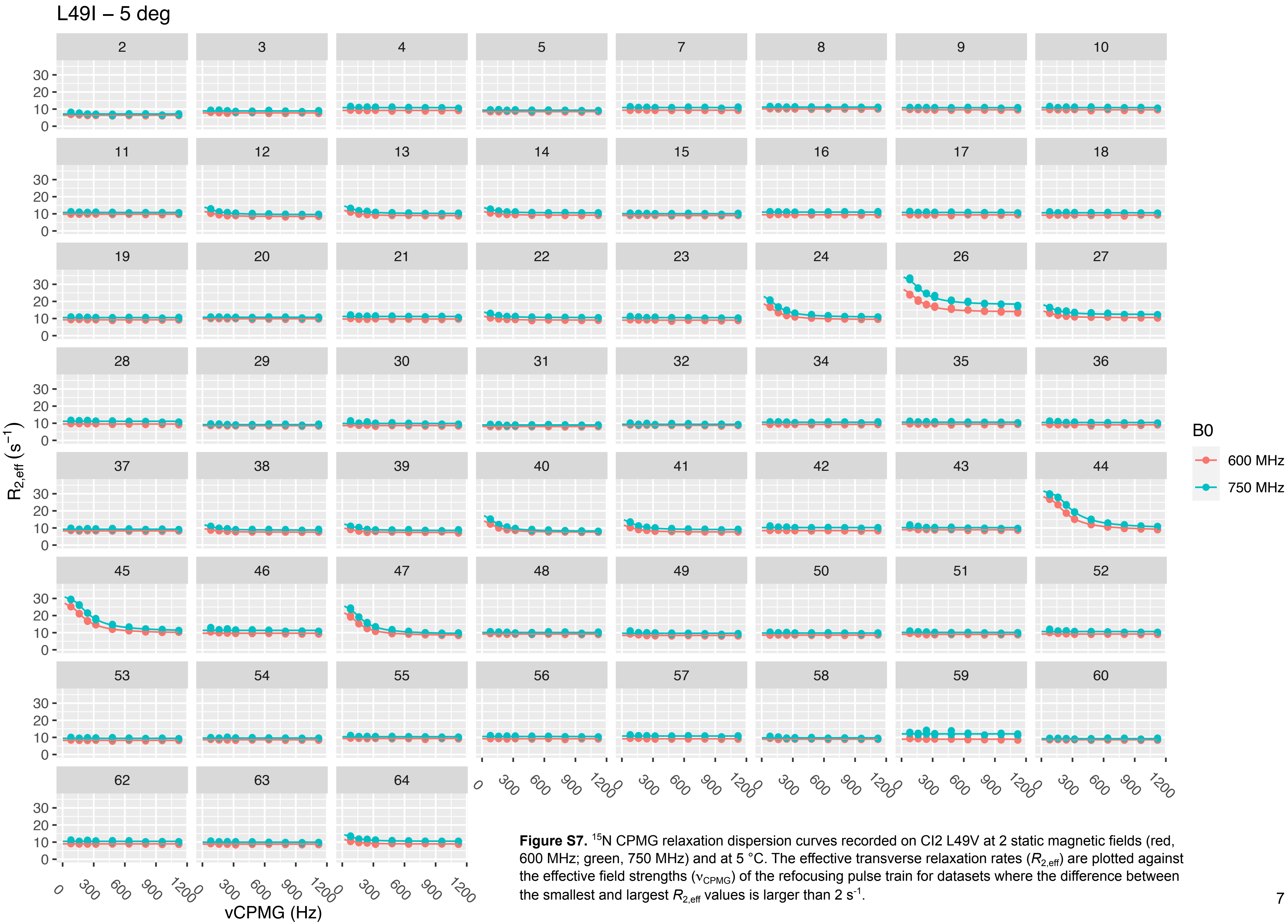


**Figure S7.** $^{15}$N CPMG relaxation dispersion curves recorded on CI2 L49V at 2 static magnetic fields (red, 600 MHz; green, 750 MHz) and at 5 °C. The effective transverse relaxation rates ($R_{2,eff}$) are plotted against the effective field strengths ($\nu_{CPMG}$) of the refocusing pulse train for datasets where the difference between the smallest and largest $R_{2,eff}$ values is larger than 2 $s^{-1}$.

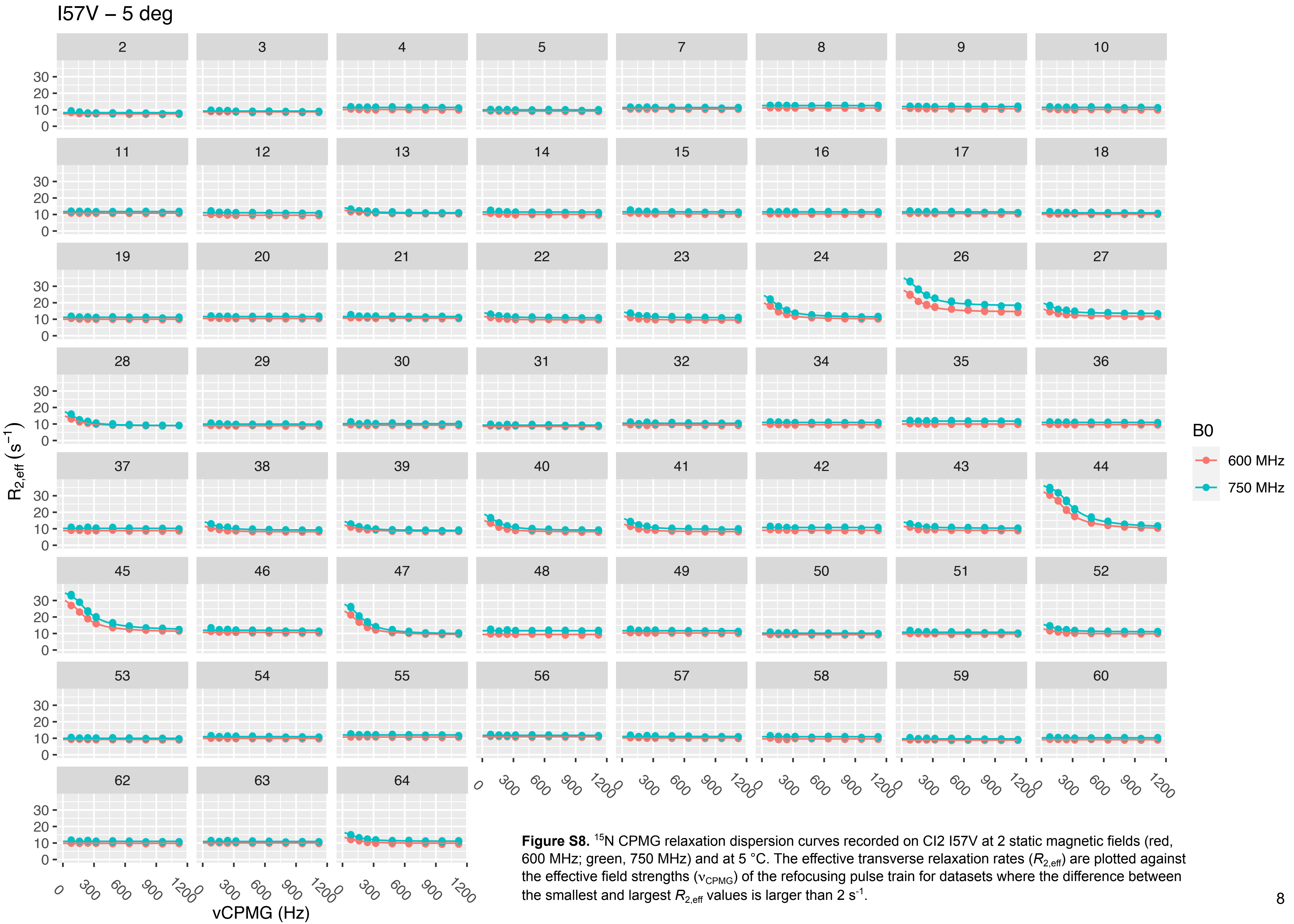


**Figure S8.** $^{15}N$ CPMG relaxation dispersion curves recorded on CI2 I57V at 2 static magnetic fields (red, 600 MHz; green, 750 MHz) and at 5 °C. The effective transverse relaxation rates ($R_{2,eff}$) are plotted against the effective field strengths ($\nu_{CPMG}$) of the refocusing pulse train for datasets where the difference between the smallest and largest $R_{2,eff}$ values is larger than 2 $s^{-1}$.

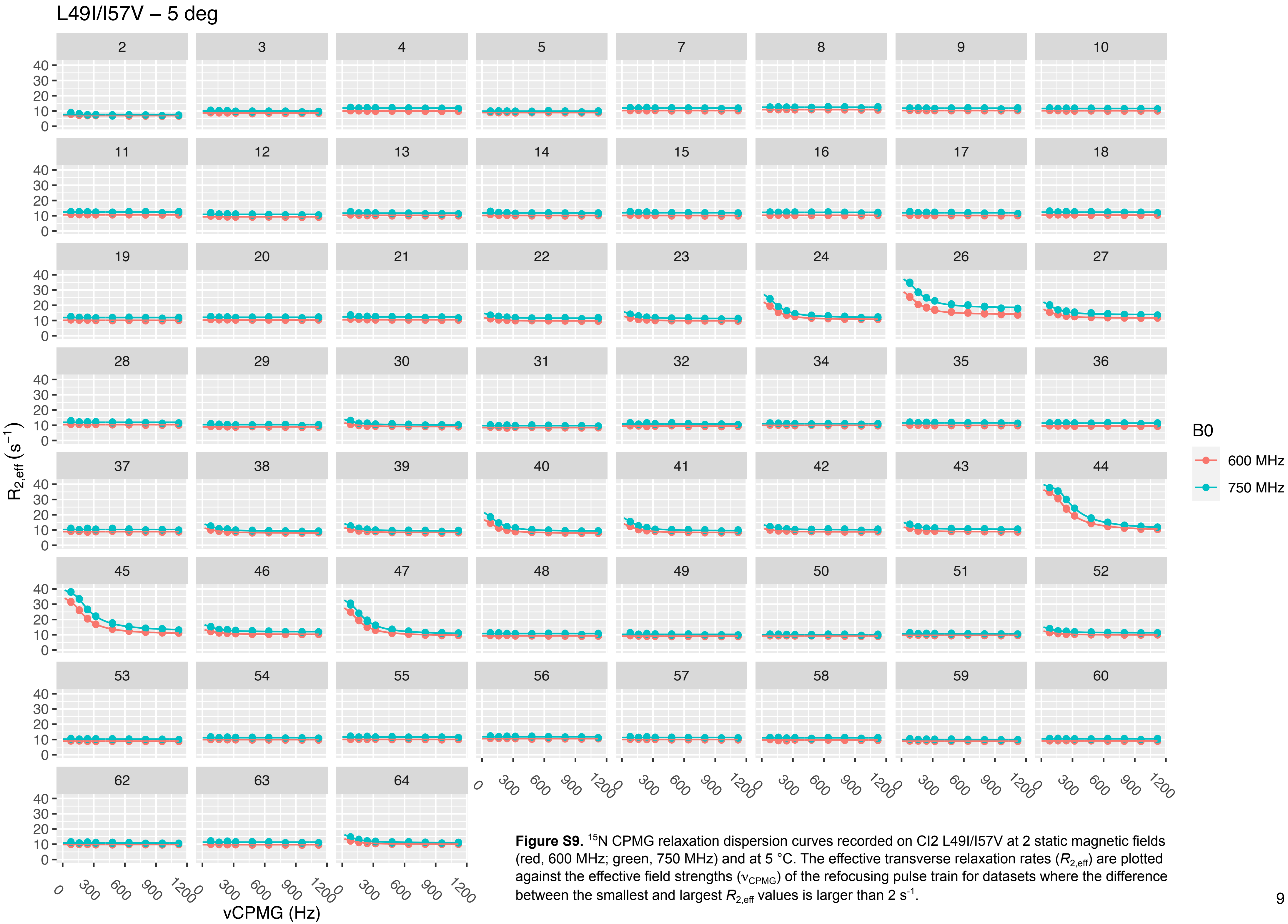


**Figure S9.** $^{15}$N CPMG relaxation dispersion curves recorded on CI2 L49I/I57V at 2 static magnetic fields (red, 600 MHz; green, 750 MHz) and at 5 °C. The effective transverse relaxation rates ($R_{2,\text{eff}}$) are plotted against the effective field strengths ($\nu_{\text{CPMG}}$) of the refocusing pulse train for datasets where the difference between the smallest and largest $R_{2,\text{eff}}$ values is larger than 2 $s^{-1}$.

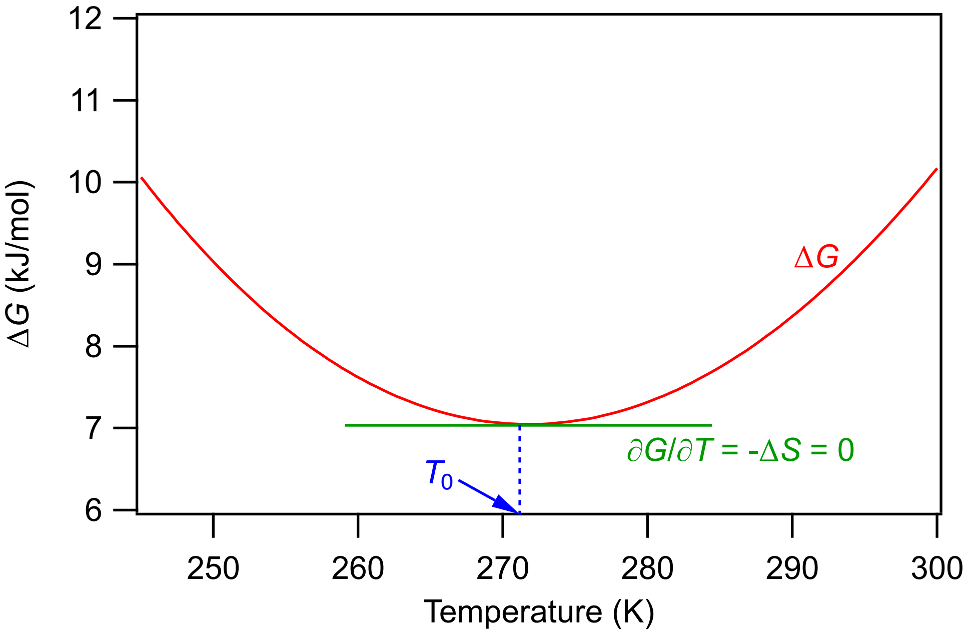


**Figure S10** Plot of $\Delta G$ vs temperature for the N to N* transition. The temperature $T_0$ where $\Delta S$ = 0 is shown. At this point $\Delta H = \Delta G$. $\Delta C_p$ is found from the curvature of $\Delta G$.

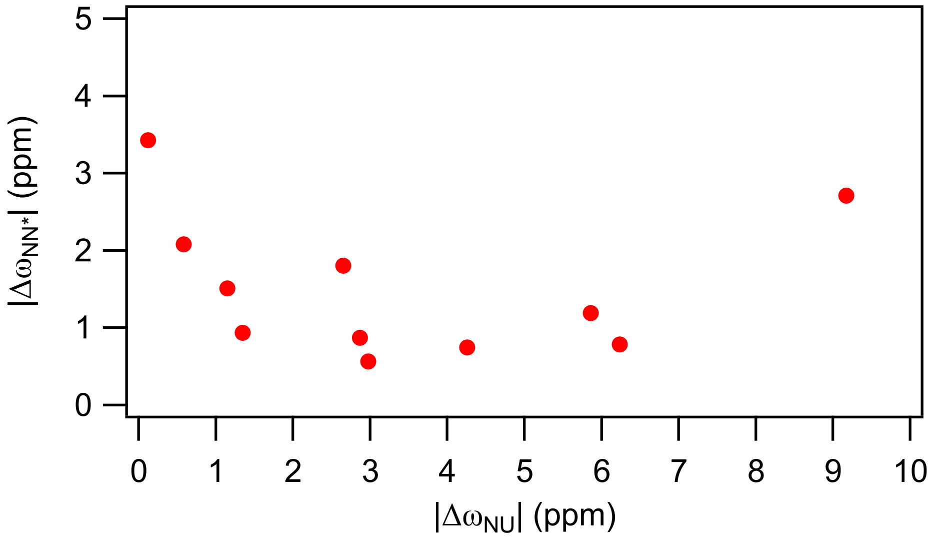


**Figure S11** The absolute chemical shift differences from relaxation dispersion measurements between the major and minor folded states plotted against the chemical shift differences between the folded and the unfolded states. The chemical shifts for the folded state were measured in a $^{15}N$-HSQC while the chemical shifts for the unfolded state were calculated with POTENCI.[1]

**Table S1.** Well-defined water molecules*

| PDB entry | Six waters | Seven waters | Eight waters | Reference |
|---|---|---|---|---|
| 1COA | x | | | (Jackson et al, 1993)[2] |
| 1LW6 | | x | | (Radisky and Koshland, 2002)[3] |
| 1TM1 | | x | | (Radisky et al, 2004)[4] |
| 1TM3 | | x | | (Radisky et al, 2004)[4] |
| 1TM4 | | | x | (Radisky et al, 2004)[4] |
| 1TM5 | | | x | (Radisky et al, 2004)[4] |
| 1TM7 | | | x | (Radisky et al, 2004)[4] |
| 1TMG | | x | | [4] |
| 1TO1 | | x | | (Radisky et al, 2004)[4] |
| 1Y1K | | | x | (Radisky et al, 2005)[5] |
| 1Y33 | | x | | (Radisky et al, 2005)[5] |
| 1Y34 | | | x | (Radisky et al, 2005)[5] |
| 1Y3B | | x | | (Radisky et al, 2005)[5] |
| 1Y3C | | x | | (Radisky et al, 2005)[5] |
| 1Y3D | | x | | (Radisky et al, 2005)[5] |
| 1Y3F | | | x | (Radisky et al, 2005)[5] |
| 1Y48 | x | | | (Radisky et al, 2005)[5] |
| 1Y4A | x | | | (Radisky et al, 2005)[5] |
| 1YPA | x | | | (Harpaz et al, 1994)[6] |
| 1YPB | x | | | (Harpaz et al, 1994)[6] |
| 1YPC | x | | | (Harpaz et al, 1994)[6] |
| 2CI2 | | x | | (McPhalen and James, 1987)[7] |
| 3CI2 | --- | No waters – NMR. | --- | (Ludvigsen et al, 1991)[8] |
| 5FBZ | | x | | (Dohnalek et al, 2016)[9] |
| 5FFN | | x | | (Dohnalek et al, 2016)[9] |
| 6QIY | | | x | (Campos et al, 2019)[10] |
| 7A1H | | | x | (Hamborg et al, 2021)[11] |
| 7A3M | | | x | (Hamborg et al, 2021)[11] |
| 7AOK | | | x | (Hamborg et al, 2021)[11] |
| 7AON | | | x | (Hamborg et al, 2021)[11] |

*Number of water molecules appearing at positions in the structure of CI2 that are similar to those shown in Figure 5C.

**Supplementary References**


1. Nielsen JT, Mulder FAA (2018) POTENCI: prediction of temperature, neighbor and pH-corrected chemical shifts for intrinsically disordered proteins. J Biomol NMR 70:141–165.

2. Jackson SE, Moracci M, elMasry N, Johnson CM, Fersht AR (1993) Effect of cavity-creating mutations in the hydrophobic core of chymotrypsin inhibitor 2. Biochemistry 32:11259–11269.

3. Radisky ES, Koshland DE (2002) A clogged gutter mechanism for protease inhibitors. Proc Natl Acad Sci USA 99:10316–10321.

4. Radisky ES, Kwan G, Lu C-JK, Koshland DE (2004) Binding, Proteolytic, and Crystallographic Analyses of Mutations at the Protease−Inhibitor Interface of the Subtilisin BPN'/Chymotrypsin Inhibitor 2 Complex. Biochemistry 43:13648–13656.

5. Radisky ES, Lu C-JK, Kwan G, Koshland DE (2005) Role of the Intramolecular Hydrogen Bond Network in the Inhibitory Power of Chymotrypsin Inhibitor 2. Biochemistry 44:6823–6830.

6. Harpaz Y, Elmasry N, Fersht AR, Henrick K (1994) Direct observation of better hydration at the N terminus of an alpha-helix with glycine rather than alanine as the N-cap residue. Proc Natl Acad Sci USA 91:311–315.

7. McPhalen CA, James MN (1987) Crystal and molecular structure of the serine proteinase inhibitor CI-2 from barley seeds. Biochemistry 26:261–269.

8. Ludvigsen S, Shen H, Kjær M, Madsen JChr, Poulsen FM (1991) Refinement of the three-dimensional solution structure of barley serine proteinase inhibitor 2 and comparison with the structures in crystals. J Mol Biol 222:621–635.

9. Dohnalek J, McAuley KE, Brzozowski AM, Østergaard PR, Svendsen A, Wilson KS Stabilization of Enzymes by Metal Binding: Structures of Two Alkalophilic Bacillus Subtilases and Analysis of the Second Metal-Binding Site of the Subtilase Family. In: Understanding Enzymes. London: Taylor & Francis; 2016. pp. 227–290.

10. Campos LA, Sharma R, Alvira S, Ruiz FM, Ibarra-Molero B, Sadqi M, Alfonso C, Rivas G, Sanchez-Ruiz JM, Garrido AR, et al. (2019) Engineering protein assemblies with allosteric control via monomer fold-switching. Nat Commun 10:5703.

11. Hamborg L, Granata D, Olsen JG, Roche JV, Pedersen LE, Nielsen AT, Lindorff-Larsen K, Teilum K (2021) Synergistic stabilization of a double mutant in chymotrypsin inhibitor 2 from a library screen in E. coli. Commun Biol 4:980.
